# Current understanding of the Alzheimer’s disease-associated microbiome and therapeutic strategies

**DOI:** 10.1038/s12276-023-01146-2

**Published:** 2024-01-04

**Authors:** Dong-oh Seo, David M. Holtzman

**Affiliations:** Department of Neurology, Hope Center for Neurological Disorders, Knight Alzheimer’s Disease Research Center, Washington University School of Medicine, St. Louis, MO 63110 USA

**Keywords:** Alzheimer's disease, Neuroimmunology

## Abstract

Alzheimer’s disease (AD) is a fatal progressive neurodegenerative disease. Despite tremendous research efforts to understand this complex disease, the exact pathophysiology of the disease is not completely clear. Recently, anti-Aβ antibodies have been shown to remove amyloid from the brain and slow the clinical progression of mild dementia by ~30%. However, exploring alternative strategies is crucial to understanding and developing more effective therapeutic interventions. In recent years, the microbiota-gut-brain axis has received significant attention in the AD field. Numerous studies have suggested that alterations in the gut microbiota composition are associated with the progression of AD, and several underlying mechanisms have been proposed. However, studies in this area are still in their infancy, and many aspects of this field are just beginning to be explored and understood. Gaining a deeper understanding of the intricate interactions and signaling pathways involved in the microbiota-AD interaction is crucial for optimizing therapeutic strategies targeting gut microbiota to positively impact AD. In this review, we aim to summarize the current understanding of the microbiota-gut-brain axis in AD. We will discuss the existing evidence regarding the role of gut microbiota in AD pathogenesis, suggested underlying mechanisms, biological factors influencing the microbiome-gut-brain axis in AD, and remaining questions in the field. Last, we will discuss potential therapeutic approaches to recondition the community of gut microbiota to alleviate disease progression. An ongoing exploration of the gut-brain axis and the development of microbiota-based therapies hold the potential for advancing AD management in the future.

## Unlocking Alzheimer’s: gut microbiota’s pivotal role in disease progression

Alzheimer’s disease is a complicated disorder involving the degeneration of nerve cells, marked by the creation of plaques and tangles in the brain, leading to mental decline. Despite much research, the precise origin and development of the disease remain unknown. This study, led by Seo and Holtzman, investigates the role of the gut microbiome (the community of microorganisms in our digestive system) in Alzheimer’s disease. The scientists conducted a narrative review, summarizing recent discoveries and discussing new questions in the field. They discovered that the gut microbiome could impact the development of Alzheimer’s disease by controlling peripheral neurotransmitters, metabolites, and immune signaling molecules. The scientists concluded that targeting the gut bacteria could potentially be a treatment strategy for Alzheimer’s disease. However, they also emphasized the need for more research to fully comprehend this intricate interaction.

This summary was initially drafted using artificial intelligence, then revised and fact-checked by the author.

## Introduction

Alzheimer’s disease (AD) is a progressive neurodegenerative disorder characterized by the early extracellular deposition of diffuse and neuritic plaques (composed of amyloid-β peptides) followed by the intracellular formation of neurofibrillary tangles (formed by hyperphosphorylated tau protein) in the brain^1^. While these aggregates represent the main pathological hallmarks of AD, the disease involves various other pathophysiological changes and processes, such as neuroinflammation, synaptic dysfunction, and metabolic dysregulation. Despite tremendous research efforts to understand the pathogenesis of the disease, the cause-and-effect relationships of the complex biological processes involved in AD are not fully understood. The lack of clarity poses significant challenges for developing effective treatments, highlighting the need for a comprehensive and multidimensional approach to tackle the disease.

The gastrointestinal (GI) tract is inhabited by numerous microorganisms, such as bacteria, fungi, and viruses, collectively called the gut microbiota (often interchangeably used with “microbiome,” which refers to the collection of genomes from all microorganisms). Recent accumulating evidence supports that dysbiosis, an imbalanced community of gut microbiota, has been linked to various brain diseases through various mechanisms regulating peripheral neurotransmitters, metabolites, and immune signaling molecules^2^.

Studies reporting altered gut microbiota composition in AD patients and animal models emerged less than a decade ago, sparking significant research in the field. Since then, numerous studies have been conducted to unravel the association between the gut microbiome and AD, and multiple mechanistic hypotheses have been suggested to explain the role of microbiota in AD, including the production of neuroactive compounds, modulation of the immune system and metabolism, regulation of the blood‒brain barrier, and involvement in the production and clearance of Aβ plaques. While this progress suggests that targeting the gut microbiota could be a potential therapeutic strategy for AD, the “microbiota-gut-AD brain axis” field is still relatively new, and there are still several gaps in knowledge that need to be addressed to fully comprehend the complex interaction between the gut microbiota and AD before treatments targeting this interaction are applied in the clinical setting.

General discussions about the relationship between the gut microbiota and AD have already been reviewed in our previous review article^3^. In this narrative review, we will briefly summarize and update the recent progress in the field and discuss emerging questions from the new observations that need further elucidation. Finally, we will discuss possibilities for disease modification by leveraging the capabilities of the gut microbiota.

## Overview of the microbiota-gut-AD brain axis

Although the idea that there is a connection between the gut and the brain has been recognized for centuries in medical history, the understanding of the specific role of the microbiota in this gut-brain axis has gained significant attention in the last few decades and become a hot topic in scientific research and public interest^4^. The exponential growth of the field is due to the development of high-throughput sequencing technologies and bioinformatics, which have enabled scientists to thoroughly characterize the composition and function of the gut microbiota^5^. This technical advancement has also opened up new avenues for studying the relationship between the gut microbiota and neurological diseases, including AD.

In 2017, Cattaneo et al. measured the stool abundance of six preselected bacterial taxa in AD patients (cognitively impaired older adults with amyloidosis) using quantitative PCR^6^. They found that AD patients show an increased abundance of proinflammatory bacteria such as *Escherichia*/*Shigella* and a decreased abundance of anti-inflammatory bacteria such as *Eubacterium rectale* compared to controls. In the same year, Vogt et al. applied the 16S rRNA gene sequencing technique to classify and identify the bacterial taxonomic composition of fecal samples from participants with and without a diagnosis of dementia due to AD. They found that the gut microbiota diversity of AD patients was significantly decreased compared to that of controls. Additionally, at the phylum level, the microbiome of AD participants shows decreased *Firmicutes* and increased *Bacteroidetes*^7^. Subsequent to these initial research endeavors, there have been similar follow-up studies characterizing the composition of the bacterial community in AD. Furthermore, recent studies have shown that individuals with mild cognitive impairment (MCI, a condition characterized by a decline in cognitive abilities that may precede the development of dementia) and even preclinical AD patients (before the onset of symptomatic AD) can exhibit distinct gut microbiota compositions compared to controls^8,9^. However, it is important to note that the details of the gut microbiome structures shown in AD are not always consistent among the studies. For example, in the gut microbiome analysis in the study by Vogt et al.^7^, the abundance of the phylum *Bacteroidetes* was increased in AD patients (97% Caucasian) compared to controls. In contrast, in another study conducted with Chinese patients, the abundance of *Bacteroidetes* was decreased in AD patients compared to controls^10^. The discrepancy might be due to several factors, including differences in study design, patient populations, lifestyles, dietary habits, and in the RNA sequencing area.

Animal studies have also supported the association between an altered gut microbiota and AD pathologies^11–13^. These studies have shown distinct differences in the microbiome composition between animal models of AD and control groups, as observed through 16S rRNA analysis of their fecal or cecum samples. However, similar to human studies, an inconsistency in the details of the gut microbiome structure among studies is also characteristic in the animal literature, even in the studies using the same type of amyloidosis animal model. For example, Brandscheid et al.^11^ observed a reduction in *Bacteroidetes* at nine weeks of age in 5xFAD mice compared to wild-type controls. In contrast, Chen et al. (2020) reported that the microbiome structures remained mostly similar between 5xFAD and WT mice at 12 weeks of age, and when animals became older, at 24 weeks of age, *Bacteroidetes, Proteobacteria, and Deferribacteres* were increased in 5xFAD mice compared to wild-type controls. The inconsistencies in gut microbiota study findings are not necessarily solely due to the high-level phylum analysis. It is not uncommon to find variations in identified families or genera associated with AD animal models across different studies. Future research efforts focusing on standardizing methodologies, employing larger sample sizes, and utilizing consistent analysis techniques can help overcome these inconsistencies and identify clear taxonomic signatures of AD-associated gut microbiota. On the other hand, the inconsistency and failure to identify clear taxonomic signatures associated with AD have prompted the field to focus on investigating the functional activities and interactions of the gut microbiota and other factors beyond taxonomic composition (e.g., metabolomics).

Numerous studies have demonstrated significant interpersonal variability in gut microbial composition and diversity^14^. The diversity among individuals regarding their gut microbiomes is substantial compared to genomic variation. While humans share approximately 99.9% similarity in their host genome, the composition and abundance of microbial species within their microbiome can vary significantly from person to person, and it is estimated that only approximately 10–20% of the microbiome (at the genera or rough species level) is shared between different individuals^15,16^. However, the field recognizes that different microbial species from distinct phylogenetic lineages can contribute to similar functional activities within the gut ecosystem^17^. This implies that even if the taxonomic compositions of two individuals’ gut microbiota are quite different, they may still exhibit similar functional activities. This recognition of “functional redundancy” underscores the importance of studying the functional activity of the gut microbiota and the metabolic potential rather than solely relying on taxonomic composition^18^. This discussion also highlights that rather than labeling specific bacterial taxa with a simplistic dichotomy as “good” or “bad,” it is more meaningful to focus on the overall composition, diversity, and stability of the microbial network and understand its functional interactions.

Driven by the need to move beyond these correlational observations and uncover the functional significance of the gut microbiota in AD pathologies, there has been a growing shift in the field to determine the direction of causality and the mechanisms involved in the microbiota-gut-AD brain axis. To test causality in the contribution of gut microbiota to the progression of AD pathologies in animal studies, mainly antibiotic treatment, germ-free/gnotobiotic models, and fecal microbiota transplantation (FMT) methods have been applied. Several studies have shown that administering a cocktail of antibiotic treatments to amyloidosis model mice (APP/PS1 mice) or raising mice in germ-free conditions reduces cerebral amyloid plaque deposition^19,20^. Furthermore, FMT using fecal samples collected from the amyloidosis animal group or introducing a specific species of bacterial taxa to AD model animals facilitates amyloid plaque deposition^21,22^.

While mounting evidence has shown that the gut microbiota regulates amyloid deposition, there was a lack of information on the contribution of the gut microbiota to tau pathology and neurodegeneration, which is strongly correlated with cognitive decline in AD. Early this year, our group assessed the hypothesis that the gut microbiota regulates tau pathology and neurodegeneration in an ApoE isoform-dependent manner. Tauopathy model mice (*P301S* tau transgenic mice) expressing human APOE isoforms (APOE3 and APOE4) were subjected to gut microbiota manipulation using two approaches: (1) being raised in germ-free conditions and (2) short-term antibiotic treatment to perturb the composition of bacterial communities. The manipulation of the gut microbiota resulted in a striking reduction in tau pathology and neurodegeneration in an ApoE isoform-dependent manner^23^. Together, these results support that gut microbiota regulates both amyloid plaque deposition and tau-mediated neurodegeneration (independent of amyloidosis).

## Underlying mechanisms and neuroinflammation

It is becoming clear that microbiota can regulate AD pathologies in model mice. However, how the gut microbiota can regulate AD pathologies in the brain that are located at a distance from the GI tract is not yet clear. The influence of the microbiota on AD might involve a combination of multiple pathways and interactions collectively contributing to AD pathologies rather than a single pathway involved in the axis. The view on the role of the microbiota in the disease has evolved in two distinct directions in recent years: (1) direct microbial infection in the central nervous system and (2) indirect pathways involving the modulation of the peripheral immune and metabolic systems. These directions represent different perspectives and emphasize distinct aspects of the gut microbiota-disease relationship.

### Direct microbial infection in the central nervous system

Microorganisms, including bacteria, viruses, fungi, and parasites, can directly cause central nervous system (CNS) infections. These infections directly affect the brain tissue or the surrounding structures, such as the meninges (protective membranes covering the brain and spinal cord) or the cerebrospinal fluid (CSF) surrounding the brain and spinal cord, and, in turn, may be able to drive or regulate AD pathologies^24^. This ‘infectious theory’ of AD was proposed approximately 30 years ago, and there have been many reports implicating diverse bacterial and viral pathogens in AD, the most frequent being Herpesviridae (particularly HSV1, EBV, and HCMV)^25,26^. This view has again been in the spotlight, as recent epidemiological reports show that two other types of herpesvirus, HHV6 and HHV7, in addition to HSV1, are associated with AD^27^. Furthermore, another epidemiological study based on Taiwan’s National Health Insurance Research Database revealed that using anti-herpetic medications to treat HSV infections was associated with a decreased risk of dementia^28^. In addition, some other bacterial species, such as *Borrelia burgdorferi*, *Chlamydia pneumonia*, *and Porphyromonas gingivalis*, have also been detected in the brain and implicated in developing AD pathologies^29–31^. However, because this perspective is yet mainly based on correlational data, it is still unclear whether microbial infection in the brain means that microbial infection activates the pathological process of AD or represents a mark of AD progression (e.g., a breakdown of pathogen protection or a reactivation of latent virus with the progression of AD pathologies). Even if the correlational data indicate that microbial infection contributes to AD, the interpretation is uncertain if the infection is the cause of AD (i.e., the origin of AD) or contributes to the progression of AD (e.g., facilitates neuroinflammation).

The infectious hypothesis proposing that a pathogen is the root cause of AD has been recently re-examined, as growing evidence supports that Aβ aggregates exhibit antimicrobial activity against certain microorganisms, including bacteria and fungi^32^. Eimer et al. tested whether a viral infection can seed and accelerate Aβ deposition to protect against pathogens in the brain^33^. They injected HSV1 into the brains of young amyloid-overexpressing model mice (5xFAD) and wild-type mice, which resulted in accelerated deposition of Aβ, and, with a lethal dose of HSV1, the transgenic mice lived longer than the controls. The study also demonstrated that Aβ oligomers inhibit HSV1 infection in vitro, suggesting that infection triggers the build-up of sticky protein plaques to protect the brain from invading pathogens. Speculatively, the progression of amyloid build-up could act as a defense mechanism, but failure to clear the amyloid can lead to an abnormal level of amyloid deposition, inflammation, and other pathologies. Despite the research progress in this view, it will be very hard to prove whether AD absolutely originates from microbial infection because amyloid deposition begins 15–20 years before symptoms appear, and it is difficult to track an individual’s microbial infection status with AD biomarker changes. Additionally, the recent preclinical studies are still based on data using an AD transgenic model animals overexpressing AD-related causative or risk genes. If microbial infection truly causes AD, researchers may need to demonstrate that a microbial infection in wild-type animals or humans drives AD-like pathologies. Regardless, it will be important to determine whether certain microorganisms can act as accelerants by increasing inflammation or AD pathology to affect the progression of the disease.

Infections can originate from multiple peripheral organs and potentially spread to the brain through various routes: the bloodstream, direct extension from nearby structures, or along nerves (e.g., the vagus nerve). It is important to note that the herpesvirus and the bacterial species discussed above are primarily found in the respiratory tract (e.g., *C. pneumonia)* or oral cavity (e.g., HSV1 and *P. gingivalis*) rather than being associated with the GI tract. However, some recent studies have indicated that CNS infections can also originate from the GI tract, contributing to AD pathologies. For example, Wu and colleagues demonstrated that the intravenous injection of *Candida albicans*, which is an opportunistic pathogenic yeast that is commonly found in the human GI tract, can establish a transient cerebritis that is marked by focal gliosis surrounding fungal cells and the deposition of both amyloid precursor protein (APP) and Aβ peptides^34^. There is also a possibility that commensal gut bacteria may be able to translocate via the vagus nerve (unpublished preprint report)^35^.

Another hypothesis is that peripheral amyloid protein might seed and promote its accumulation in the brain due to its retrograde transport to the CNS via the vagal nerve or blood. This discovery has led to the idea that some bacteria produce extracellular amyloid fibers called curli, which also adopt a beta-sheet structure^36^. Interestingly, recent studies showed that intra-GI administration of Aβ_1–42_ oligomers in wild-type mice caused cerebral beta-amyloidosis^37,38^. However, further studies are needed to determine whether bacterially produced amyloid actually contributes to AD pathologies or peripheral Aβ deposition with AD progression and then contributes to brain amyloidosis later on^11^. Additionally, the underlying mechanism for enteric Aβ-induced amyloidosis in the brain is still unclear.

### Indirect pathway modulating the peripheral immune and metabolic systems

Currently, the most plausible hypothesis for mechanistic explanations in the field is that neuroinflammation mediates the interaction between gut microbiota and the progression of AD pathologies. Reactive astrogliosis and microgliosis are prominent pathological features in the AD brain. Primarily, glial cells provide support and protection to neurons, clearing dead cells and foreign particles to maintain homeostasis in the brain. However, disease-associated abnormal activity of glial cells disrupts homeostatic cellular networks in the brain and accelerates AD progression. That is, abnormally activated glial cells may propagate Aβ toxicity, lead to Aβ accumulation, or release proinflammatory cytokines and reactive oxygen species, which are harmful to neurons and facilitate tau pathology, and further excessive inflammation leads to neuronal damage and disease progression (for a more detailed review^39,40^). Remarkably, mounting evidence from animal studies has demonstrated that the gut microbiota regulates the maturation and function of glial cells. Erny and colleagues, in 2015, showed that the depletion of microbiota by raising mice in germ-free conditions or administering antibiotic treatment resulted in microglia being in immature states with a reduced response to viral infections. They also found that microbially produced metabolites called short-chain fatty acids (SCFAs) are involved in this interaction^41^. Interestingly, there exists a sex-specific pattern in the influence of the gut microbiota on microglial maturation during development^42–44^. Likewise, a recent study showed that primary cultured astrocytes could exhibit sex-specific differential responses to SCFA treatment^45^.

Corroborating these findings, several studies investigating the role of gut microbiota in AD have demonstrated that gut microbiota manipulation in AD animal models resulted in morphological and gene expression changes in glial cells accompanying the reduction in AD pathologies (i.e., Aβ deposition, tau pathology, and neurodegeneration)^20,23,46,47^. Furthermore, the reduction in Aβ deposition by antibiotic-induced gut microbiota manipulation was prevented by depleting microglia using a colony-stimulating factor 1 receptor (CSF1R) inhibitor^48^.

However, the precise mechanisms underlying the activation of brain innate immunity are still being investigated. The gut microbiota helps maintain immune homeostasis by regulating the balance between proinflammatory and anti-inflammatory responses^49^. Primarily, immune activation is an essential defense mechanism of the host to protect against pathogens. However, an excessive inflammatory response leads to an increase in circulating cytokines and recruitment of leukocytes, including effectors of cellular adaptive immunity (i.e., B or T lymphocytes), which infiltrate the tissue or release cytokines that activate brain innate immunity. Eventually, these processes can accelerate neuroinflammation^50,51^. Studies using AD animal models have also demonstrated that the manipulation of the gut microbiota reduces multiple peripheral cytokines related to macrophages and adaptive immune cells^52^. Our recent study using an animal model of tauopathy also demonstrated that meningeal *γδ T cells, plasmacytoid dendritic cells, and NK cells are gut microbiota dependent*. These immune cells release cytokines, such as interleukin-17, interferon type-I response, and others, which may affect neuroinflammation in the brain, even without brain infiltration^23^. Human studies have also shown that AD patients show altered peripheral immune signatures^50,53^. However, these studies have identified differences in peripheral cytokine profiles, and it remains unclear which immune agents are directly involved in the activation of brain innate immunity in the context of AD. Further research is necessary to establish a direct link between peripheral cytokine changes and the activation of innate immunity in the brain.

While the neuroinflammation pathway currently occupies significant attention as a mechanistic explanation in this domain, nonglial mechanisms may also be involved in the interaction. Amyloidosis model mice (APP/PS1) raised in germ-free conditions showed an increase in amyloid clearing enzymes such as neprilysin degrading enzyme (NPE) and insulin-degrading enzyme (IDE) compared to conventionally raised mice, which may affect Aβ deposition^19^. Other biological factors may amplify the influence of the gut microbiota. For example, age and genetic factors may disrupt gut permeability and blood–brain barrier (BBB) integrity, which accelerates the entry of circulating inflammatory agents and pathogens into the brain, driving excessive activation of brain innate immunity^54,55^. In addition, microbial metabolites such as SCFAs have also been found to modulate autophagic activity, a cellular process that plays a crucial role in maintaining cellular homeostasis by clearing and recycling cellular debris, damaged proteins, and organelles^56,57^. Dysfunction in autophagy machinery and alterations in the regulation of autophagy-related genes have been reported in AD brains^58^. It is worth exploring the possibility that circulating SCFAs regulate autophagic activities in AD brains^59^.

## Gut microbiota-derived metabolites: SCFAs and others

One of the fundamental processes involved in the interaction between the gut microbiota and the host is the production and exchange of metabolites. These small molecules, which can be directly derived from bacteria, bacterial metabolism of dietary substrates, or the modification of host molecules, influence the host’s immune and metabolic systems^60^.

The most extensively investigated metabolites in this field are SCFAs, which are organic acids with a chain length of fewer than six carbon atoms, and they are primarily produced through the fermentation of dietary fibers by gut bacteria. As discussed above, SCFAs regulate the maturation and function of brain innate immunity^41,45^. Glia does not express SCFA receptors, so SCFAs might regulate glia indirectly via peripheral immune cells that express SCFA receptors. In addition, SCFAs can regulate the production of cytokines by peripheral immune cells such as neutrophils, macrophages, and dendritic cells (DCs) and the proliferation and differentiation of T cells and B cells^61–64^. These regulations may transform glial cells. It is also possible that SCFAs directly regulate glial functions. Erny and colleagues found that microglia take up acetate (one of the major SFCAs) that has entered the CNS, and the acetate modulates microglial functions via epigenetic and mitochondrial metabolic mechanisms^46^.

The general perception of SCFAs is that they play a beneficial role in human health, such as providing energy to the cells and supporting the integrity of the intestinal barrier^65^. However, the picture is more complex in regard to the impact of SCFAs on neurological diseases. While studies have shown that SCFAs improve recovery from several neurological disorders, such as multiple sclerosis, stroke, and traumatic brain injury, recent studies using animal models of AD and Parkinson’s disease suggest that SCFAs can facilitate neuroinflammation and disease progression in the absence of the gut microbiota^23,66–68^. Even within the AD field, the effects of SCFAs are not always consistent, as some literature indicates that one SCFA supplement, sodium butyrate, alleviates Aβ deposition^69,70^. It is unclear what led to this inconsistency, but it is possible that each SCFA, such as acetate, propionate, and butyrate, could have distinct effects and mechanisms of action in various contexts (the detrimental effect in the AD model was observed with mixed SCFA supplementation) or that the same SCFA-driven peripheral immune response may have a differential role in the pathology of each disease^71,72^. In addition, SCFAs can exert different effects on various physiological processes based on their levels within the body. The same doses of mixed SCFAs (sodium propionate, 25.9 mM; sodium butyrate, 40 mM; sodium acetate, 67.5 mM) were used across the above studies. Future studies need to test the effect of different doses of SCFAs on the development of AD pathologies. Last, host age at SCFA supplementation can be an important factor to consider. When we administered SCFA supplements to tauopathy model animals, we observed the promotion of hippocampal gliosis in old mice (35 weeks old) but not in young mice (15 weeks old)^23^.

In addition to SCFAs, other metabolites have been implicated in AD clinical studies, such as LPS, trimethyl-amine N-oxides (TMAO), tryptophan, and bile acids^50,60,73–75^. Although the metabolic dysregulation of these factors has been observed in AD patients, the specific role of these metabolites in AD has yet to be well investigated. Further investigation is necessary to comprehensively understand the role of metabolites beyond SCFAs in AD.

## Host genetic variants and sex differences

The extent to which host genetic variation shapes the composition of the microbiome, as opposed to environmental factors, is an ongoing debate and research in microbiome science. While many studies suggest that the human gut microbiome is itself shaped by a wide variety of environmental factors, including diet, lifestyle, geography, medication use, and exposure to pathogens, others have reported that host genetic variation is a strong regulator of the host microbiome^76,77^.

The apolipoprotein E (APOE) genotype is the most prevalent genetic risk factor for neuropathology and AD. Recent studies have examined the gut microbiota composition of humans or model animals carrying different human APOE alleles. An analysis of the gut microbiome of APOE2, APOE3, or APOE4 carriers revealed that SCFA-producing bacterial families, such as *Ruminococcaceae* and *Lachnospiraceae*, were more abundant in APOE2 carriers and less abundant in APOE4 carriers than in APOE3 carriers in a stepwise fashion (i.e., APOE2 > APOE3 > APOE4)^78,79^. All details in comparing microbiome structures between APOE isoforms are not always consistent. For example, Tran et al. reported that *Lachnospiraceae* was increased in APOE4 mice compared to APOE3 mice^80^. This discrepancy might be due to the different environmental exposures or diets among studies, but it also implies that environmental factors interact with genetic factors in microbiome shaping. We recently evaluated whether the gut microbiota regulates tau-mediated neurodegeneration by interacting with *APOE* isoforms using antibiotic-induced gut microbiota perturbation in an animal model of tauopathy. Gut microbiota manipulation was neuroprotective against tau pathology and neurodegeneration. However, the effectiveness was limited to males, and remarkably, there were greater effects in the presence of ApoE3 than ApoE4^23^. This differential isoform effect with gut microbiota manipulation might be due to the combination of differential peripheral and brain innate immune responses with APOE4, and eventually, ABX failed to protect against tau-mediated neurodegeneration. However, future studies need to precisely investigate what tissue, cell type, and pathway are affected by these APOE and microbiome interactions. Additionally, the exact mechanisms by which APOE alleles may differentially modulate the gut microbiome still need to be fully understood. Different APOE alleles may have varying effects on immune function and inflammation (e.g., differential macrophage response and other peripheral immune cells), which can, in turn, influence the gut microbiome composition^81^. Additionally, as the primary role of APOE is involved in lipid metabolism and the transport of lipids, differential lipid metabolism or alterations in gut barrier function associated with specific APOE alleles could affect the gut environment and microbial communities.

In addition to host genetic variation, sex differences have been recognized as important factors in the context of the gut microbiome and its response to antibiotics in AD model animals. In the studies discussed above, antibiotic-induced gut microbiota perturbation reduced AD pathologies in males but not females. This same pattern of results was observed across multiple studies using both amyloidosis and tauopathy animal models^20,23^. The mechanisms whereby biological sex may differentially modulate antibiotic-associated pathological outcomes are not known. There is a sex-specific microglial maturation pattern that interacts with the gut microbiota during development^42–44^. Additionally, it is known that there are sex differences in the gut microbiome structure and immune response to pathogens; females often exhibit stronger innate immune and T-cell proliferation/antibody responses than males^82,83^. However, it is unclear what exact differences account for the nonresponse to antibiotics in females in these studies. Last, sex hormones such as estrogen play a role in modulating the immune system and neuroinflammatory responses in females^82^. The hormonal differences between males and females might influence how the gut microbiota responds to antibiotics and subsequently impact AD pathologies. Combining ovariectomy and antibiotics may have implications for the differential response to antibiotics in AD pathologies, or only sex-specific genetic factors may influence the susceptibility or resilience of the brain to neuroinflammation, amyloid-beta aggregation, and tau pathology.

## Beyond the gut and bacteria

Although we have mainly focused on the microbiota present in the GI tract in this review, recent studies have indicated that the microbiota in locations other than the GI tract, such as the lung and oral microbiome, may play a role in the development or progression of AD. For example, Maurer et al. identified several oral bacterial strains, including *P. gingivalis*, more frequently in AD patients with periodontitis than controls. Additionally, another study showed that oral infection with *P. gingivalis* in an amyloidosis animal model impairs cognitive function and increases the deposition of AD-like plaques^31^. The lung microbiome has also gained increasing attention concerning various neurological diseases. A recent paper found that LPS-producing lung microbiota resulted in disease exacerbation using an animal model of multiple sclerosis, a T-cell-mediated autoimmune disease of the central nervous system^84^. The current methods to manipulate the microbiota, such as germ-free conditions or specific antibiotic treatments, can affect the microbiota in other organs and tissues throughout the body in addition to the GI tract. The impact of these manipulations on non-GI microbiota is an important consideration when interpreting results.

Additionally, in the microbiome-AD field, most studies mainly focus on bacteria and less on viruses and fungi. One reason might be that most studies used bacterial 16 S rRNA gene sequencing techniques for microbiome analysis up to now (therefore, the terms “microbiota” or “microbiome” generally refer to the bacterial community in this review). In addition, the proportional abundance of other microorganisms, such as fungi, protozoa, archaea, and viruses, is lower than that of bacteria, and these other microorganisms have thus received less attention. However, even though their abundance is low, they may still affect AD pathology directly or indirectly by interacting with the bacterial communities—all types of microorganisms interact with each other and form a dynamic network with their host. Therefore, microbiome-AD research needs to expand into investigations of fungal communities (mycobiome), viral communities (virome), and other microorganisms. In the future, functional analyses, such as metagenomic, metatranscriptomic, or metabolomic analyses, will provide insights into the functional activities, interactions, and pathways within the microbiota that contribute to AD.

## Therapeutic strategies

**Fig. 1:**
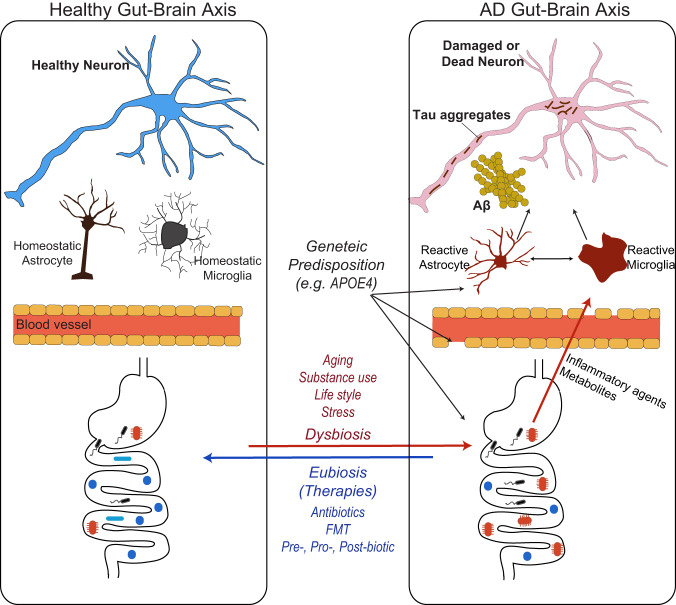
Schematic diagram of the contribution of gut microbiota to AD pathologies and microbiota-based AD interventions. Commensal microorganisms are essential for a healthy brain (left). However, multiple factors, including aging processes, dietary changes, and drug/alcohol consumption, can result in the unbalanced composition of gut microbial communities, influencing peripheral metabolism and immunity (right; red line). These biological processes ultimately regulate brain innate immunity and the progression of AD pathologies. Additionally, genetic risk factors (e.g., the presence of an ApoE4 allele) may contribute to or influence the presence of more disease-associated microbiota, the reactivity of brain innate immunity, and blood–brain barrier (BBB) integrity (black line). The blue line in the figure represents the therapeutic potential of putative microbiota-based interventions. Reconditioning the unbalanced gut microbiota using fecal microbiota transplantation (FMT), dietary fiber prebiotics, or probiotics limits the contribution of dysbiosis to AD progression, preventing the shift toward detrimental peripheral inflammation, metabolism, and neuroinflammation.

As we discussed, the gut microbiota regulates multiple aspects of AD pathologies, including Aβ accumulation, tau pathology, and neurodegeneration, potentially through effects on neuroinflammation and metabolic homeostasis. As supported by recent findings, the modulation of the gut microbiota has emerged as a promising avenue to slow AD progression^85^. Several approaches are being explored to modulate the gut microbiota in AD. These include antibiotics, FMT, prebiotics, probiotics, postbiotics, and some emerging biotechnologies, such as *encapsulants and bacteriophages*. These interventions aim to restore a healthy balance of the gut microbial community and alleviate AD pathologies and symptoms (Fig. 1).

### Antibiotics

Antibiotics are primarily designed to treat bacterial infections by targeting and killing harmful bacteria or inhibiting their growth. In microbiome research, antibiotics are often utilized as a tool to manipulate the microbial community in a broad and nonspecific manner for experimental purposes. The objective is typically to assess the overall function of the microbiota in a particular disease or biological process rather than to be used in a direct clinical application. Because the nonspecific eradication of both pathogenic and beneficial bacteria can drive dysbiosis and other adverse effects^86^, antibiotic treatments might not be ideal for clinical applications in AD patients.

Additionally, because the antibiotics in preclinical studies were often applied in early experimental animals’ lives or during the presymptomatic period, it is still being determined whether antibiotic use after the AD symptomatic period effectively alleviates AD pathology and symptoms. In future studies, applying an experimental design corresponding to therapeutic purposes (i.e., testing the effect of antibiotics after the onset of pathologies begins) will allow us to assess the possibility of using antibiotics for AD. Interestingly, a recent epidemiological study supports this possibility. Rakusa et al. analyzed public health insurance data in Germany to investigate the relationship between antibiotics for systemic use and dementia. They found a decreased likelihood of dementia with preceding antibiotic use^87^.

### Fecal microbiota transplantation

FMT is a procedure that involves transferring fecal material from a healthy donor into the GI tract of a recipient. FMT aims to restore the imbalanced gut microbiota to a more balanced and beneficial gut microbiota composition in the recipient and can be a potential therapeutic tool for AD.

Kim et al. demonstrated that the transplantation of the fecal microbiota from wild-type mice into transgenic model mice expressing *APP*, *PSEN1*, and *MAPT* transgenes (ADLP^APT^) ameliorated the formation of Aβ plaques and neurofibrillary tangles, glial reactivity, and cognitive impairment^22^. Additionally, there is an interesting case report with an 82-year-old male AD patient who suffered from *Clostridioides difficile* infection (CDI). The patient underwent a single FMT infusion using stool from the patient’s 85-year-old wife as a donor, who was intellectually acute. Even 2 months after FMT, the patient showed rapid improvement in AD symptoms^88^. Likewise, other quantitative studies also showed that FMT significantly improved clinical symptoms in AD patients compared to controls, supporting that FMT might effectively delay cognitive decline in AD patients^89,90^.

### Prebiotics, probiotics, and postbiotics

Antibiotics are used to treat a broad spectrum of potentially fatal bacterial infections in a nonspecific way. The nonspecific action of antibiotics can have unintended consequences on the gut microbiota, including a disruption of the normal microflora, leading to potential nutrient shortages and the possibility of opportunistic pathogens taking over. In consideration of this, an alternative approach to recondition the imbalanced gut microbiota is the introduction of beneficial bacteria to the GI tract. This approach involves the use of probiotics (live microorganisms that, when administered in adequate amounts, confer a health benefit on the host; e.g., *Bifidobacterium* and *Lactobacillus*), prebiotics (special fibers that promote the growth of beneficial bacteria; e.g., inulin), and postbiotics (substances produced by beneficial bacteria during their growth, which can directly benefit our health; e.g., SCFAs).

The potential benefits of probiotics in AD are still in the early stages of investigation due to our limited understanding of the complex relationship between microorganisms and AD. However, several preclinical and limited clinical studies have explored the effects of probiotics (e.g., SLAB51, *Bifidobacterium breve*, and *Akkermansia muciniphila*) in animal models and small groups of Alzheimer’s patients^59,91–94^. These studies have shown some promising results, such as improvements in cognitive function and a reduction in amyloid-beta plaques, which potentially occur through the anti-inflammatory effects of probiotics.

An indirect way to recondition the gut microbiota is by providing the necessary nutrients for beneficial bacteria to thrive. Dietary fiber prebiotics are carbohydrates found in plant-based foods that human enzymes cannot digest in the small intestine, but once they reach the large intestine, gut microbiota selectively metabolize them. This microbiota-associated metabolic process contributes to various health conditions by producing metabolites, synthesizing vitamins, and modulating the immune system. In addition, these metabolic processes also lead to changes in the gut microbiota composition, promoting the growth of beneficial bacteria, influencing microbial diversity, and affecting host health^95,96^. However, little is known about the impact of dietary fiber on AD progression.

Although the general conception is that high-fiber consumption is beneficial for health, recent preclinical animal studies discussed above suggest that SCFAs, produced through the microbial breakdown of dietary fiber, drive neuroinflammation and AD pathologies^23^. While some recent studies have shown the beneficial effects of SCFAs in neurological preclinical animal studies, it is still possible that a high level of dietary fiber consumption results in the overproduction of SCFAs and is detrimental to AD patients rather than beneficial^68,71,72^. Therefore, a deeper mechanistic understanding of how dietary fiber interacts with the progression of tau pathology and neurodegeneration will be critical for establishing whether a targeted dietary change in AD patients is safe and effective in improving outcomes. Similarly, SCFAs have also gained positive public perception due to their potential health benefits. However, in the context of AD, it is too early to know what to recommend or to regard postbiotics because their specific effects on AD pathologies are still being investigated, and more research is needed to fully understand the mechanisms of action and potential therapeutic applications of dietary fiber prebiotics, probiotics, and postbiotics in AD. A recent study demonstrated that sodium oligomannate, a mixture of acidic linear oligosaccharides derived from *brown algae*, reduces metabolite-driven peripheral infiltration of immune cells into the brain, inhibits neuroinflammation, and reverses cognitive impairment by reconditioning the gut microbiota in an amyloidosis animal model. This study further supports the emerging idea that gut microbiome modulation using prebiotics can be a novel strategy to slow AD progression^85,97^.

## Conclusion

In the last decade, thanks to the advancements in microbiome analytic technology and bioinformatics, there has been significant progress in establishing the role of microbiota (particularly gut microbiota) in AD. Pioneering studies have demonstrated that gut microbiota regulates the development of AD pathologies. However, as many studies have confirmed, the microbiota in our body can affect almost all aspects (i.e., physiology, immunology, and metabolism) of the host, and microbiota communities are affected by many biological factors, such as genes and sex. Therefore, it is challenging to identify a single pathway involved in the “microbiota-gut-AD brain” axis.

Nevertheless, the research progress in the microbiome-AD field supports the notion that modulating the gut microbiome could be a promising strategy to slow AD progression. Treatments such as antibiotics, prebiotics, probiotics, postbiotics, FMT, and other strategies need to be explored for their ability to restore the balance of the gut microbiota and potentially mitigate AD pathology. However, before these applications can be effectively utilized, a comprehensive understanding of the mechanistic pathways connecting the gut microbiota to AD pathology is crucial to ensure the safety and efficacy of these interventions.

Clinical trials investigating the modulation of the gut microbiota in AD are still in the early stages, and more evidence is needed to determine the effectiveness of these interventions. Challenges in this field include the complexity of the gut microbiota, interindividual variability, and the requirement for standardized protocols and rigorous study designs. Long-term studies are necessary to assess the sustained effects of microbiota modulation and its impact on AD progression. In addition, developing new techniques for modulating the gut microbiota more selectively would be beneficial for advancing therapeutic approaches: Approaches involving microbial encapsulation (enclosing microbial cells within a protective polymeric matrix), bacteriophages (selectively targeting and eliminating specific bacteria), microbial enzyme modulators (modulating the activity of specific microbial enzymes to slow down or prevent certain biochemical reactions), and other bioengineered microbes to produce beneficial metabolites are emerging in this field. These approaches could lead to controlled-release and targeted interventions to efficiently restore microbial balance and function in the gut.

In conclusion, while the modulation of the gut microbiota shows promise as a potential strategy to slow AD progression, further research is needed to understand the underlying mechanisms in the interaction between microbiota and AD and to establish effective interventions.
